# The roles played by highly truncated splice variants of G protein-coupled receptors

**DOI:** 10.1186/1750-2187-7-13

**Published:** 2012-09-01

**Authors:** Helen Wise

**Affiliations:** 1 School of Biomedical Sciences, Faculty of Medicine, The Chinese University of Hong Kong, Shatin, Hong Kong, SAR, China

**Keywords:** Alternative splicing, Cell signaling, G protein-coupled receptors, Truncated splice variants

## Abstract

Alternative splicing of G protein-coupled receptor (GPCR) genes greatly increases the total number of receptor isoforms which may be expressed in a cell-dependent and time-dependent manner. This increased diversity of cell signaling options caused by the generation of splice variants is further enhanced by receptor dimerization. When alternative splicing generates highly truncated GPCRs with less than seven transmembrane (TM) domains, the predominant effect *in vitro* is that of a dominant-negative mutation associated with the retention of the wild-type receptor in the endoplasmic reticulum (ER). For constitutively active (agonist-independent) GPCRs, their attenuated expression on the cell surface, and consequent decreased basal activity due to the dominant-negative effect of truncated splice variants, has pathological consequences. Truncated splice variants may conversely offer protection from disease when expression of co-receptors for binding of infectious agents to cells is attenuated due to ER retention of the wild-type co-receptor. In this review, we will see that GPCRs retained in the ER can still be functionally active but also that highly truncated GPCRs may also be functionally active. Although rare, some truncated splice variants still bind ligand and activate cell signaling responses. More importantly, by forming heterodimers with full-length GPCRs, some truncated splice variants also provide opportunities to generate receptor complexes with unique pharmacological properties. So, instead of assuming that highly truncated GPCRs are associated with faulty transcription processes, it is time to reassess their potential benefit to the host organism.

## Introduction

G protein-coupled receptors (GPCRs) constitute the most abundant gene family in most animal species [1] and are the target of at least 50% of marketed drugs [2]. The number of GPCRs varies widely among different species, with the elephant having almost three times as many genes as the 1265 found in humans [3]. The question then is, “does this matter?” GPCRs are not only receptors for light and for biologically active chemicals, but also respond to stimuli associated with smell and taste. Therefore, the higher number of GPCR genes in the elephant may just reflect its greater reliance on its sense of smell. However, simply counting the number of genes in a genome greatly underestimates the number of gene products actually produced because of a process known as alternative splicing. By overlooking the number and variety of GPCR splice variants, we may misinterpret the fine-tuning options for controling cell signaling activity in health and disease. Furthermore, it has been proposed that GPCR oligomerization also provides a way to increase the number of receptor entities with a limited number of genes [4,5], and even apparently non-functional highly-truncated splice variants of GPCRs can generate functionally active GPCRs by heterodimerization [6,7]. To add further to this complexity of GPCR signaling, even though individual cell types typically express more than one hundred different GPCRs, the most highly expressed GPCRs are not necessarily those which have been targeted therapeutically [3]. So even if splice variants are expressed less well than their wild-type counterparts, this does not automatically mean they are of little consequence. Because dysregulation of GPCR activity contributes to many different pathophysiological processes [8], we still need to carefully consider the specific role of each GPCR and its splice variants. Alternative splicing generates GPCRs with variations in specific structural domains, and in this review we will focus on the highly truncated splice variants which represent approximately 50% of all GPCR isoforms studied to date. We will show that generalizations assuming highly truncated GPCRs are non-functional may be an oversimplification, and we will discuss how GPCR dimerization between wild-type receptors and their splice variants has added a new level of regulation to the complex process of cell signaling. This review will also focus on some unique splice variants offering insights into the physiological role of these GPCR isoforms.

### Alternative splicing of genes

**Figure 1:**
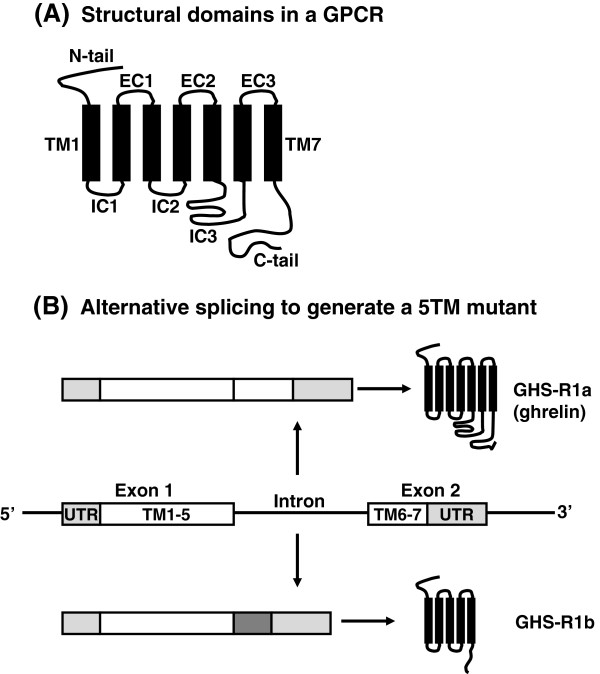
**Alternative splicing of a GPCR.** ( **A**) The structural domains of GPCRs are annotated as N-tail (amino terminus), EC1-3 (extracellular loop domains 1–3), TM1-7 (transmembrane domains 1–7), IC1-3 (intracellular loop domains 1–3) and C-tail (carboxy terminus).( **B**) Generation of a 5TM mutant of the ghrelin receptor results from failure to remove the intron between the two coding exons. An alternative stop codon and a polyadenylation signal within the intron (dark box) are used to produce a C-terminal truncated GPCR form that is unable to bind to growth hormone secretagogues [14].

Alternative splicing is a major mechanism for modulating the expression of genes and enables a single gene to increase its coding capacity, allowing the synthesis of several structurally and functionally distinct protein isoforms [9,10]. In humans, a typical primary transcript, or precursor to mRNA (pre-mRNA), contains seven or eight introns and eight or nine exons, which together average more than 27,000 nucleotides in length [11]. The removal of introns (pre-mRNA splicing) is carried out by spliceosomes, ribonucleoprotein complexes that recognize the exon-intron junctions and catalyse the removal of introns and subsequent joining of exons. There are several different types of alternative splicing [11,12] which allows a single primary transcript to yield different mature RNAs; an example of intron retention is given in Figure 1. It is not unreasonable to assume that a large fraction of all human mutations affect splicing activity [11], and that the ratio of isoforms will ultimately affect normal cellular function. Any differences in the activities or amounts of general splicing factors and/or gene-specific splicing regulators during development or in different tissues will cause differential patterns of splicing. This means that transcriptome analysis, rather than genome analysis, is needed to assess the real impact of alternative splicing on human diseases [9]. Splice variants do not just represent the end-products of gene transcription, they can also change the regulation of this process. For example, intron 4 of the human neuropeptide Y receptor (Y_1_) efficiently promotes the increased production of Y_1_ but this function is missing in the splice variant [13].

### GPCR trafficking – ER retention of highly truncated splice variants

GPCRs are seven-transmembrane (7TM) domain receptors (Figure 1) which are trafficked through the biosynthetic pathway to the cell surface in a tightly regulated mechanism with multiple steps and a stringent quality control system to ensure correct GPCR folding and targeting. Association of GPCRs with accessory proteins or chaperones are a key step for the forward trafficking through the endoplasmic reticulum (ER) and Golgi [15]. The life of GPCRs begins in the ER where they are synthesized, folded and assembled [16,17]. During their migration to the cell surface, GPCRs undergo post-translational modifications to attain mature status. While knowledge of the structure-function relationship of GPCRs has been extended by recent high-resolution structural studies of β_2_-adrenoceptors [18] and muscarinic M_2_ receptors [19], little is known of features influencing overall stability of the highly truncated mutant GPCRs. In some cases, alternative splicing generates mutant transcripts which are simply too faulty to be expressed; splice variants of the human endothelin (ET_B_) receptor do not appear to be translated, or the products are quickly degraded, presumably because of their instability [20]. Nevertheless, the preferential production of this null function ET_B_ by RNA editing/splicing has been proposed to underlie the etiology of Hirshsprung disease [20], so even the most unstable GPCRs can impact on overall physiology.

Because the ER forms part of the cellular quality control machinery where functionally inactive mutant GPCRs can be prevented from expression at the cell surface [21], it is not unusual to find highly truncated GPCRs retained in the ER (see Table 1). Conditions such as X-linked nephrogenic-diabetes insipidus, familial hypocalciuric hypercalcemia, familial glucocorticoid deficiency or hypogonadodotropic hypogonadism are associated with mutations in GPCRs which result in intracellular retention in the ER or Golgi compartments [22]. For this reason, alternative splicing which generates truncated GPCRs which are retained in the ER may likely to be associated with pathological conditions. Significant ER retention is increasingly seen as the norm because as much as 50% of all newly synthesized protein fails to meet ER exit quality control criteria [23]. Much of the work in this field has highlighted the importance of C-terminal tails that can influence the efficiency of ER exit as well as the internalization and endosomal sorting of GPCRs [24]. Examples are human gonadotrophin-releasing hormone (GnRH) receptors, luteinizing hormone/choriogonadotropin (LH/CG) receptors [25], rhodopsin [26] and vasopressin V_2_ receptors [27].

**Table 1:** GPCR splice variants with altered or deleted transmembrane domains

**Receptor**	**Splicing consequence**	**General properties of splice variant**	**Dom/neg?**	**Refs.**
α_1A-_adrenoceptor	Truncated 6TM mutants.	No cell surface expression.	Yes	[28]
Calcitonin receptor (CT receptor)	Truncated 6TM mutant.	Rabbit CTRΔe13 is poorly expressed on the cell surface and fails to activate cell signaling.	Yes	[29]
Chemokine receptor 5 (CCR5)	Severely truncated mutants. Ccr5Δ32 is a 5TM mutant.	Natural mutants of human chemokine receptor CCR5 lacking the last 3 or 5 TMDs are non-functional. Ccr5Δ32 complexes with CCR5 and retains CCR5 in the ER, thus reducing cell surface expression.	Yes	[30,31]
Dopamine receptor (D_3_)	D3nf is a truncated 5TM mutant.	Human D3nf mutant has a punctuate perinuclear distribution and does not bind DA-ligands.	Yes	[32,33]
GABA_B_ receptor (GABA_B_R1)	GABA_B_R1c has an additional 31 amino acids in TM5.		No	[34]
Gastric inhibitory polypeptide receptor (GIP )	Truncated 4TM mutant.	This inactive mutant receptor inhibited GIP signaling and decreased cell surface expression by retaining WT receptor in the ER.	Yes	[35]
Ghrelin receptor (or GHS-R1a)	GHS-R1b is a truncated 5TM mutant.	GHS-R1b is a non-signaling splice variant of GHS-R1a.	Yes	[36]
Gonadotropin-releasing hormone receptor (GnRH receptor)	Truncated 5TM mutant.	5TM variant of human GnRH receptor shows decreased cell surface expression, no ligand binding and no signal transduction.	Yes	[37]
Growth hormone-releasing hormone receptor (GHRH-R)	Truncated 5TM mutant.	The mutant GHRH-R cannot transduce GHRH signals.	Yes	[38]
Histamine receptor (H_3_)	6TM-rH_3_R is a truncated 6TM mutant.	Several splice variants of rat H_3_R do not bind agonist, have an intracellular localization and co-expression with WT receptor decreases cell surface expression and functional responses.	Yes	[39]
Histamine receptor (H_4_)	hH_4_R(302) lacks 88 amino acids from TMD2 to TMD4. hH_4_R(67) is a ~ 1.5TM truncated mutant.	These human H_4_R splice variants were localised predominantly intracellularly when expressed in recombinant cells. No ligand binding or cell signaling detected.	Yes	[40]
Leukotriene B_4_ receptor (BLT_1_)	LTB4R-AS1 lacks TMD2 and part of ECL1 (39 amino acid deletion).	Both isoforms of human BLT_1_ (LTB4R) are expressed in human airway smooth muscle cells.	Yes	[41]
	LTB4R-AS2 is the 3TMD to C-terminus (lacks 100 amino acids).			
Luteinizing hormone receptor (LH receptor)	Truncated 5TM mutant.	The 5TM mutant of human LH receptor binds ligand (limited) but has no signaling activity.	Yes	[42]
μ-opioid receptor (μ-OR)	Truncated 6TM mutant.	A 6TM μ-OR variant in mice identified ligands lacking the traditional side effects of classical opiates but maintaining analgesic property.	No	[6]
Motilin receptor	GPR38-B is a truncated 5TM mutant.		?	[43]
Neurokinin 2 receptor (NK_2_)	An ICL2-TM4 deletion mutant.	NK_2_β splice variant is poorly expressed on the cell membrane and is non-signaling.	Yes	[44]
Neuropeptide Y receptor (Y_1_)	A truncated 5TM mutant.	The putative hY_1_-related 5TM accessory protein encoded by the non-spliced hY1 mRNA is not involved in facilitating hY_1_ production.	No	[13]
Neurotensin receptor (NTS_2_)	Truncated 5TM mutant with long tail.	Rat vNTS_2_ is functionally active and can heterodimerize with NTS_2_.	No	[45]
Nociceptin receptor (NOP)	Truncated 4TM mutant.	The rat truncated NOP receptor is localised to cell membranes but is non-functional.	Yes	[46]
Prostaglandin F_2α_ receptor (FP)	A 6TM truncated mutant (FP_s_)	hFP_S_ is functionally inactive and highly expressed in the perinuclear region.	Yes	[47]
	A truncated mutant (PTGFR-v2).	No distinct functional role identified.	No	[48]
Somatostatin receptor (sst_5_)	Human sst5TMD5 is a 5TM truncated mutant.	Identified novel truncated but functional human sst_5_-variants; present in normal and tumoral tissues.	No	[49]
	Human SST5TMD4 is a 4TM truncated mutant.			
	Murine sst5TMD4 is a 4TM truncated mutant.	Three murine variants were functional to mediate ligand-selective-induced variations in Ca^2+^ and cAMP despite being truncated and displayed a preferential intracellular distribution.	No	[50]
	Murine sst5TM2 is a 2TM truncated mutant.			
	Murine and rat sst5TM1 has just TM1.			
Vasopressin receptor, subtype 2 (V_2_)	V_2_b is a truncated 6TM mutant.	V_2_b is retained in the ER where its C-terminus can be either intracellular or extracellular.	Yes	[51]

For the GnRH receptor, a single change in net charge is sufficient to tip the balance in favour of the ER and diminish GnRH receptor available at the plasma membrane [52]. Conn *et al.* (2006) [22] have proposed that the apparent inefficiency of the GnRH receptor must have evolved under strong and convergent evolutionary pressure, suggesting there must be a strong advantage to generating an inefficiently produced receptor which is highly susceptible to cause a mutational disease. Whether such unexpected evolutionary pressure also exists for the ER-retained highly truncated GPCRs is unknown.

### GPCR dimerization and its influence on GPCR trafficking

It is becoming apparent that many GPCRs form homo and/or hetero-dimers or higher order oligomers and that dimerization could both positively and negatively regulate GPCR cell surface targeting [53]. But it is important to realize that some functional consequences that are proposed to originate from heteromeric receptor interactions may also be observed due to intracellular crosstalk between signaling pathways of non-associated GPCRs [54]. Heterodimerization between different GPCR subtypes can significantly modify functional characteristics of the individual protomers, included subcellular localization, ligand binding co-operativity and proximal signaling [55].

That homodimerization was a prerequisite for cell surface targeting was first identified with the β_2_-adrenoceptor [21]. A few examples of other GPCRs with constitutive dimers/oligomers which form during biosynthesis are: α_1D_-adrenoceptors (in complex with β_2_-adrenoceptors) [56]; GABA_B_ receptors [57]; ghrelin receptors [58]; gonadotrophin hormone (LH/hCG and FSH) receptors [59,60]; neurotensin NTS_1_ and NTS_2_ receptors [61]; oxytocin receptors [62]; and vasopressin V_1a_ and V_2_ receptors [62]. Collectively, most data suggests that receptor oligomers are preassembled in the ER and ‘walk hand-in-hand’ to the cell surface [54].

The impact of GPCR heterodimerization on cell surface targeting is perhaps best exemplified by GABA_B_ receptors [63]. The GABA_B_ receptor (GABA_B_R1) did not couple effectively to expected signaling pathways until co-expressed with GABA_B_R2 which allowed GABA_B_1R to escape from the ER [64]. Expression of the GABA_B_R1 subunit on the cell surface was prevented through a C-terminal retention motif which needed to be masked by heterodimerization with GABA_B_R2 [64]. In contrast, the heterodimerization of α_1A_- and α_1D_-adrenoceptors primarily involves the hydrophobic core of these receptors as deletion of the C-terminal domains did not affect cell surface expression [65]. While there is evidence for specific heterodimerization between wild-type GPCRs and highly truncated isoforms in the ER [58], the structural motifs responsible for heterodimerization with truncated splice variants and subsequent ER retention are unknown. It is currently possible to rescue GPCRs retained in the ER using chemical or pharmacological chaperones [66], and this presents an interesting approach to overcome the dominant-negative effect of the highly truncated GPCR splice variants.

Evidence of heterodimerization of full length GPCRs *in vivo* has been harder to obtain, [67], but examples are available associated with pathophysiological conditions: angiotensin-II (AT_1_) – bradykinin receptor complexes in preeclampsia [68]; the μ-opioid receptor and α_2A_-adrenoceptor complex in depression and opioid addiction [69]; the adenosine A_2A_ – dopamine D_2_ receptor complex in Parkinson’s disease [70]; the κ-δ-opioid receptor complex in analgesia and drug abuse [71] and the metabotropic glutamate (mGlu_2_) – 5-hydroxytryptamine (5-HT_2A_) receptor complex in schizophrenia [72,73]. As highly truncated splice variants are relatively hard to detect *in vivo*, definite proof of the existence of their heterodimerization with full length GPCRs is harder to obtain (see next section).

To address the issue of whether or not truncated GPCRs are functionally active, we need to consider if ER-retained GPCRs are capable of signaling activity. There is a growing body of evidence supporting the hypothesis that endocytosed receptors can activate specific signal transduction pathways [74]. But what about signaling from the ER before trafficking of receptors to the cell surface? Studies with β_2_-adrenoceptor have shown that during its trafficking through the ER/Golgi, this GPCR is already pre-associated with its G proteins and effector enzyme (adenylyl cyclase 2) in a signaling complex [75]. Another example of a GPCR with activity in the ER is the ghrelin receptor. Constitutive activation of extracellular signal-regulated kinases (ERK1/2) can be detected in the ER of HEK293 cells expressing ghrelin receptors [58]. Both observations support the idea that GPCR signaling can be activated in the ER, but whether or not GPCR signaling in the ER is fundamentally different from GPCR signaling at the cell surface remains to be determined.

For non-constitutively active GPCRs, there is the issue of access of agonist to the receptor when it is retained in the ER. Nuclear metabotropic glutamate (mGlu_5_) receptors are orientated with their ligand binding domain inside the nucleoplasmic reticulum, and an active transport process is used so that glutamate can access their binding sites in this intracellular compartment [76]. As the ER is contiguous with the nuclear membrane [77], it is possible that similar systems exist to facilitate cell signaling of other GPCRs from this intracellular location. For GPCRs such as the thyroid-stimulating hormone (TSH) receptor, activation of adenylyl cyclase in different subcellular compartments regulates different cellular functions [78]. Ultimately, one should not necessarily assume that ER-retained GPCRs and their splice variants will be non-signaling, or that they will serve the same function as receptors on the cell surface.

### Splice variants of GPCRs - Introduction

Previously, it was assumed that splice variants of ligand-gated channels were common, whereas each GPCR was far more likely to be a single product of its gene [79]. Thus, initial estimates suggested that approximately 90% of mammalian GPCRs were intronless in their open reading frame, which compares with approximately 35% for other cell surface receptors [80]. Access to additional databases and bioinformatics studies now indicates that perhaps only 50% of mammalian GPCR genes are intronless and do not require post-transcriptional processing [81]. The relative absence of introns in human GPGRs could indicate that most GPCR genes were derived from a single intronless common progenitor relatively recently in evolutionary history [80]. Because intronless genes do not require post-transcriptional splicing, we might expect them to be transcribed more efficiently [80], but this in turn would decrease diversity. After all, GPCR genes that do possess introns can undergo alternative splicing, generating GPCR subtype isoforms that may differ in their pharmacological, signaling and regulatory properties. Splice variants of GPCRs were often dismissed as the consequence of ‘leaky transcription’ and hence deemed physiologically irrelevant [82]. Despite this, we see that splice variants of GPCRs are not uncommon, and that formation of heterodimers and/or retention of functionally-active GPCRs in different subcellular compartments greatly increases the complexity of GPCR signaling.

The largest group of splice variants per GPCR relates to the C-terminus [79,82] but distinct differences can exist between human and rat/mouse orthologs such as the EP_3_ receptor [83], making it essential to understand the relationship between human and non-human GPCRs before inferring mechanisms based on non-human receptor studies. When genomic libraries were first prepared, the possibility of alternative gene splicing was generally ignored [79], with the first clear evidence in 1989 pertaining to the rat dopamine D_2_ receptor [84]. GPCR splice variants often show differential distribution among many tissues and brain regions, consistent with cell-specific control of transcription and splicing [82], and alternative versions of a gene transcript might be necessary for different tissue types or at different stages of life [80]. Thus, alternative splicing serves as a molecular tool to introduce more diversity into gene expression and this may have been generated as a more economical alternative to gene duplication during evolution [82].

Given that GPCRs can exist as dimers or oligomers, we might expect to observe dominant-negative effects in some heterozygous individuals, which could relate to defective routing of the complex formed between the wild-type and mutated receptors to the plasma membrane [85]. Some clinical evidence described in obese patients with mutations in melanocortin (MC_4_) receptor supports this concept, but, with a few exceptions, expression of the disease in heterozygous individuals is usually mild or absent [86].

Alternative splicing of the GPCR superfamily in human airway smooth muscle has recently been demonstrated to diversify the complement of receptors [41]. Out of the 434 GPCRs detected in airway smooth muscle, 192 GPCRs had, on average, five different expressed receptor isoforms. There was no apparent relationship between ‘wild-type’ expression levels and the occurrence of a particular splicing event. Of note in this study was the relatively low expression levels of GPCRs such as the M_3_-muscarinic receptor, β_2_-adrenoceptor, and receptors for leukotrienes and prostanoids which are already targeted therapeutically. Therefore, a low expression level of GPCRs does not necessarily equate to functional insignificance.

### The dominant-negative effect of GPCR splice variants with altered or deleted transmembrane domains, generating highly truncated mutant receptors

**Figure 2:**
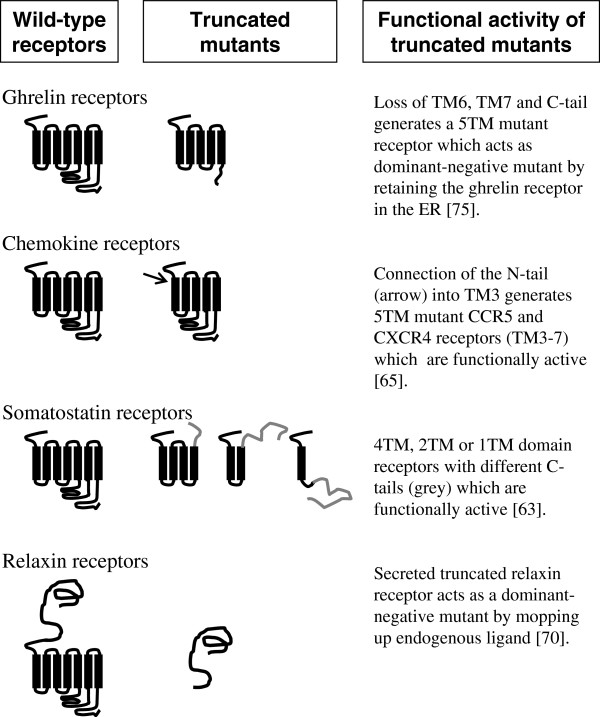
Schematic representation of the structural relationship between wild-type and truncated GPCRs.

The majority of truncated GPCR splice variants act as dominant-negative mutations, but there are always the exceptions, with truncated human somatostatin (sst_5_) variants being surprisingly functional [50]. These sst_5_ variants maintain the same N-terminal region as full-length sst_5_ receptors, but have different, shorter C-terminal tails with 4, 2 or 1 TM domains (see Figure 2 and Table 1) [49,50]. Similarly, mutant chemokine receptors (CCR5 and CXCR4) comprising merely the N-terminus and TM3 to TM7 domains, i.e., lacking TM1 and TM2 (Figure 2), can still function normally when expressed in HEK293 cells [87]. So, the full 7TM domains are definitely not a prerequisite for effective cell signaling. But, if the GPCR had lost TM3, we would expect this to severely compromise GPCR signaling as the highly conserved E/DRY motif in the cytoplasmic surface of TM3 has been shown to be essential for coupling of the GPCRs such as vasopressin V_2_ receptors, α_1B_-adrenoceptors and angiotensin-II (AT_1A_) receptors to G proteins [88] by forming an ‘ionic lock’ with a glutamate residue in TM6 [89]. Thus, the functional significance of specific TM domains is highly dependent on the particular GPCR.

For GPCRs with relatively large N-terminal ectodomains, alternative splicing can generate truncated receptors lacking the N-terminal domain. Cleaving the N-terminal domain from the remaining TM domains generates a more specific dominant-negative effect due to the released N-terminal domain (Figure 2). Hence, soluble non-membrane anchored ‘receptors’ are seen for the corticotrophin-releasing factor receptor (CRF_2_) [90], metabotropic glutamate (mGlu_6_) receptor [91] and the relaxin receptor (RXFP1) [92]. These secreted proteins are proposed to act as biological modifiers of endogenous ligand-stimulated activity by mopping up endogenous ligand and preventing activation of the respective wild-type receptors.

#### Association of truncated GPCRs with pathophysiological conditions

Although there are some exceptions (see sst_5_ receptors above), splice variants resulting in truncated GPCRs of just 4 – 5 TM domains tends to generate functionally inactive mutant receptors. In Table 1 we have 22 examples of truncated GPCRs and the majority have no cell surface expression and no cell signaling activity. These mutant GPCRs are retained in an intracellular compartment, typically the ER and frequently function as dominant-negative mutants of the wild-type receptors. Retaining the wild-type receptors in the ER can have significant functional consequences, dependent on the constitutive role of the wild-type GPCR and/or its role in mediating responses to pathogenic organisms. For example, the chemokine receptor 5 (CCR5) functions as a co-receptor for HIV-1 infection, thus mutations allow for a protective role against HIV infection [30]. The 5TM ccr5Δ32 splice variant complexes with and retains CCR5 in the ER, so the *ccr5Δ32/ccr5Δ32* genotype has been linked with a phenotype that is “highly” protected from HIV-1 infection, whereas the *CCR5/ccr5Δ32* genotype confers only “relative” protection [31]. As seen with CCR5 and its 5TM splice variant, formation of specific heterodimers between truncated GPCRs and their associated wild-type receptors has been identified with the dopamine D_3_ receptor [32,33], ghrelin receptor mutant polypeptide (GHS-R1b) [36] and histamine H_4_ receptor [40], but the association with a specific pathology remains speculative. The intracellular localization pattern of the other 5TM mutants listed in Table 1 strongly suggests they too form heterodimers with corresponding wild-type GPCRs resulting in decreased cell surface expression of these receptors. Unfortunately, the absence of antibodies capable of distinguishing wild-type and truncated GPCRs *in vivo* continues to hamper developments in this area.

While intracellular retention of wild-type GPCRs can arise by heterodimerization with 5TM mutants, there are other means to ultimately decrease expression of wild-type GPCRs. In schizophrenia, there is a decrease in dopamine D_3_ receptor mRNA but no change in 5TM D3nf mutant [93]. The enhanced D3nf-specific splicing of D_3_ pre-mRNA in schizophrenia may lead to this decreased expression of D_3_ mRNA, and thus a decrease in D_3_ receptor protein [94]. To generate D3nf mRNA by alternative splicing requires a rare splice event capable of recognising just 98 nucleotides as an alternative intron within a larger 2675 nucleotide exon [94]. Despite its rarity, this splicing event clearly occurs.

The differential expression of truncated splice variants is perhaps the norm [40,47,49,95,96], but there are always exceptions. The somatostatin (sst_5_) receptor splice variants showed no marked differential expression, but they were differentially regulated by changes in hormone/metabolic environment in a tissue- and ligand-dependent manner [50]. An altered hormone/metabolic environment resulting from a high-fat diet changes the ratio of expression of gastric inhibitory polypeptide (GIP) receptor and its 4TM mutant in mice [35]. The relative reduction of truncated GIP receptor expression may be involved in the hypersensitivity of GIP and hyperinsulinemia in these obese mice. Similarly, the expression of luteinizing hormone (LH) receptor and its 5TM truncated splice variant (LHd) in the corpus luteum varies at different stages of the ovarian cycle [96]. This profile of independent regulation for sst_5_, LH and GIP and their splice variants in response to different physiological conditions contrasts with the prostaglandin F_2α_ (FP) receptor splice variants. FP receptor splice variants are differentially expressed in endothelial cells and in highly vascularised tissues [47], but no distinct role for PTGFR-v1 or PTGFR-v2 has been identified in relation to an altered cellular environment associated with pregnancy [48]. Ultimately, the functional role of each pair of wild-type GPCR and its truncated splice variant has to be studied under both normal and pathophysiological conditions, but proving a specific role for these highly truncated receptors will remain problematic when there is insufficient sequence difference between splice variant and wild-type GPCR mRNA to selectively knock down the splice variant using siRNA techniques.

#### Association of truncated GPCRs with constitutively active counterparts

The ghrelin receptor (GHS-R1a) and histamine H_3_ and H_4_ receptors are constitutively active and therefore can function independent of agonist [39,40,97], and dominant-negative effects have been observed with their truncated splice variants (see Table 1). The human H_4_ splice variant is differentially expressed in CD34+ cord blood-derived eosinophils and mast cells, but the functional consequence of heterodimerization with wild-type H_4_ is presently unknown [40]. For the ghrelin receptor, we see marked effects of its truncated 5TM splice variant (GHS-R1b) on constitutive activation of phospholipase C, with no effect on activation of ERK1/2 [36,58,98]. Bioluminescence resonance energy transfer (BRET) studies suggested that heterodimers of GHS-R1a/GHS-R1b are concentrated in the ER, whereas homodimers of GHS-R1a are more uniformly distributed throughout the cell [58]. It has been proposed that conflicting findings concerning the role of ghrelin in different tissues could be linked to the presence of this ghrelin receptor polypeptide GHS-R1b [14,99]. There is no correlation between the expression of GHS-R1a mRNA and GHS-R1b mRNA in different tissues [95], and the factors regulating the expression of ghrelin receptor isoforms are unknown. The constitutive activity of the ghrelin receptor attenuates apoptosis via a protein kinase C-dependent process *in vitro*[100], and because GHS-R1b has a dominant-negative effect on ghrelin receptor function, any changes which increase GHS-R1b relative to GHS-R1a would be predicted to have a functional effect.

For an individual receptor, it is unclear to what degree such ligand-independent receptor signaling is present in the *in vivo* situation and consequently whether the constitutive signaling is of physiological relevance [101]. Some clues can be gained from other natural mutations of the ghrelin receptor and the melanocortin (MC_4_) receptor. Two families have been identified in which short stature is segregated with a *GHSR* mutation (Ala204Glu) that is characterized by substantially decreased basal activity of the receptor [102], possibly due to decreased expression *in vivo*[103]. Of the individuals who were identified as heterozygous for this mutation, not all had short stature. This observation is compatible with codominant transmission of the trait, with incomplete penetrance of the phenotype [102]. The constitutive activity of the MC_4_ receptor is maintained by its N-terminal domain, and mutations in the N-terminal lead to loss of basal activity and functional defects [104]. Obesity-associated mutations in the N-terminal domain of MC_4_ decrease its constitutive activity, which suggests that in addition to the agonist-mediated activation of MC_4_, this constitutive activity is also required for the maintenance of the anorexigenic catabolic state and the prevention of obesity in humans [104]. Together, these observations suggest that the physiological mechanisms controlled by ghrelin receptors and MC_4_ would be highly sensitive to the level of their basal activity. Since co-expression of ghrelin receptors and GHS-R1b in HEK293 cells dramatically decreases constitutive activation of phospholipase C [36,98], and that this effect may be physiologically relevant, we have proposed that alternative splicing of genes for other constitutively active GPCRs will also dramatically affect cell signaling and functional activity.

#### Generation of receptor signaling complexes with altered pharmacology

Despite this dominant property of truncated GPCRs acting as dominant-negative mutants, there are examples where some functional activity is maintained. A 6TM μ-opioid receptor (MOR-1) variant in mice can identify ligands which lack the traditional side effects of classical opiates but maintain significant analgesic properties [6], and a 5TM somatostatin (sst_2_) receptor also retains functional activity and generates a receptor displaying an altered pharmacology [49]. A large series of splice variants of μ-OR (MOR) have been isolated from mice, rats, and humans with similar splicing patterns [6]. Most MOR-1 knock-out mice with a disruption of exon 1 were unresponsive to morphine, but mice with a series of MOR-1 variants generated from a second, upstream promoter associated with exon 11 had quite different pharmacology. These exon 11-associated variants lacking exon 1 are 6TM truncated variants, lacking the first TM domain of MOR-1 encoded by exon 1. One proposed partner for this inactive 6TM MOR-1 variant is the nociceptin (NOP) receptor [6]. By partnering of the mutant MOR-1 with NOP, the resulting heterodimers have a unique pharmacology which may provide valuable therapeutic targets.

The ghrelin receptor polypeptide (GHS-R1b) is another example of a functionally inactive truncated GPCR which can generate novel pharmacology by heterodimerization with a related GPCR. In this example, GHS-R1b can heterodimerize with the neurotensin receptor 1 (NTS_1_) to provide a receptor capable of responding to neuromedin U [7]. Treatment of non-small cell lung cancer cells with siRNA for GHS-R1b or NTS_1_ suppressed their growth in response to autocrine production of neuromedin U [7]. Indeed, when the ghrelin receptor splice variant GHS-R1b was first discovered [14], there was a rash of papers profiling GHS-R1a and GHS-R1b mRNA expression in human tumours, attempting to link altered expression with the state of malignancy [105-110]. It might be fruitful instead to look for potential novel partners for truncated splice variants in cells and tissues where conventional molecular tools have failed to compliment pharmacological identification of receptor subtypes.

## Conclusions

By analogy with GPCR heterodimerization as a means to expand the repertoire of cell signaling [111], we can see that the availability of splice variant protomers will similarly influence the cell signaling capacity of GPCRs. It is well established that the majority of GPCRs are desensitized, internalized and down-regulated by constant or repeated exposure to agonist. And, it is now established that trafficking of GPCRs to the cell surface is a highly regulated process, and heavily influenced by the expression of highly truncated splice variants. Is it possible that expression of these splice variants allows fine-tuning of these processes? Is it possible that activation of a GPCR ‘auto regulates’ expression of its spliced isoforms or regulates the expression of other GPCR isoforms? At the present time, it is premature to answer these intriguing questions. The expectation of dominant-negative mutants to inactivate cell signaling by retaining the functional wild-type GPCR in the ER is perhaps an oversimplification because retention in the ER does not necessarily mean a loss of cell signaling activity. Furthermore, splice variants with 5TM or 6TM domains can be functionally active and present unique pharmacology by forming heterodimers with 7TM domain receptors. We could start by looking for potential novel partners for truncated splice variants in cells and tissues where conventional molecular tools have failed to compliment pharmacological identification of receptor subtypes. Currently, the incidence and association of splice variants with disease is an area of intense interest as a means to better target GPCR-dependent therapies. However, the association between GPCR polymorphisms and clinical disease are currently too weak to allow clinically meaningful predictions of GPCR variants and their relationship to disease onset or progression, or in drug responses [112]. Perhaps now we need to concentrate on treating each GPCR as a unique entity and thoroughly assess its activity profile in relation to the co-expression of splice variants in a cell-dependent and a time-dependent manner.

## Competing interests

The author declares that they have no competing interests.

## Authors’ contributions

HW is the sole author.
